# Lower genomic stability of induced pluripotent stem cells reflects increased non-homologous end joining

**DOI:** 10.1186/s40880-018-0313-0

**Published:** 2018-07-25

**Authors:** Minjie Zhang, Liu Wang, Ke An, Jun Cai, Guochao Li, Caiyun Yang, Huixian Liu, Fengxia Du, Xiao Han, Zilong Zhang, Zitong Zhao, Duanqing Pei, Yuan Long, Xin Xie, Qi Zhou, Yingli Sun

**Affiliations:** 1 Key Laboratory of Genomic and Precision Medicine, China Gastrointestinal Cancer Research Center, Beijing Institute of Genomics, Chinese Academy of Sciences, Beijing, 100101 P. R. China; 2 University of Chinese Academy of Sciences, Beijing, 100049 P. R. China; 3 State Key Laboratory of Stem Cell and Reproductive Biology, Institute of Zoology, Chinese Academy of Sciences, Beijing, 100101 P. R. China; 4 The Key Laboratory of Regenerative Biology, Guangdong Provincial Key Laboratory of Stem Cell and Regenerative Medicine, South China Institute for Stem Cell Biology and Regenerative Medicine, Guangzhou Institutes of Biomedicine and Health, Chinese Academy of Sciences, Guangzhou, 510530 P. R. China; 5 CAS Key Laboratory of Receptor Research, the National Center for Drug Screening, Shanghai Institute of Materia Medica, Chinese Academy of Sciences, Shanghai, 201203 P. R. China

**Keywords:** Genomic stability, DNA damage repair, iPSCs, ESCs

## Abstract

**Background:**

Induced pluripotent stem cells (iPSCs) and embryonic stem cells (ESCs) share many common features, including similar morphology, gene expression and in vitro differentiation profiles. However, genomic stability is much lower in iPSCs than in ESCs. In the current study, we examined whether changes in DNA damage repair in iPSCs are responsible for their greater tendency towards mutagenesis.

**Methods:**

Mouse iPSCs, ESCs and embryonic fibroblasts were exposed to ionizing radiation (4 Gy) to introduce double-strand DNA breaks. At 4 h later, fidelity of DNA damage repair was assessed using whole-genome re-sequencing. We also analyzed genomic stability in mice derived from iPSCs versus ESCs.

**Results:**

In comparison to ESCs and embryonic fibroblasts, iPSCs had lower DNA damage repair capacity, more somatic mutations and short indels after irradiation. iPSCs showed greater non-homologous end joining DNA repair and less homologous recombination DNA repair. Mice derived from iPSCs had lower DNA damage repair capacity than ESC-derived mice as well as C57 control mice.

**Conclusions:**

The relatively low genomic stability of iPSCs and their high rate of tumorigenesis in vivo appear to be due, at least in part, to low fidelity of DNA damage repair.

## Background

Embryonic stem cells (ESCs) are pluripotent and could differentiate into all types of somatic cells [1]. ESCc have enormous potential in the treatment of a variety of diseases, but their clinical application has been limited by ethical controversy. In 2006, Yamanaka and colleagues overexpressed four transcription factors (*Oct4*, *Sox2*, *c*-*Myc* and *Klf4*) in mouse somatic cells and obtained ESC-like pluripotent stem cells, termed induced pluripotent stem cells (iPSCs) [2]. iPSCs resemble ESCs in morphology, gene expression profile, epigenetic status and in vitro differentiation capacity. The development of iPSCs raises new hope for personalized clinical therapy [3–5].

The four transcription factors (*Oct4*, *Sox2*, *c*-*Myc* and *Klf4*) that are critical for the production of iPSCs are frequently overexpressed in various cancers, and mice derived from iPSCs are prone to develop tumors [6–9]. Although only a small population of transformed cells with genetic mutations is likely to develop into tumors [10], the genomic instability of iPSCs is a major concern that could produce huge impact on their eventual clinical use [11–16].

One possible explanation for the observed greater genomic instability of iPSCs is alterations in the fidelity of DNA repair pathways. Double-stranded DNA breaks, for example, can be repaired via homologous recombination (HR) with high fidelity, or via non-homologous end joining (NHEJ) with lower fidelity [17–20]. In the current study, we examined whether iPSCs differ from other types of pluripotent cells in their ability to perform these types of DNA repair. Briefly, ionizing radiation was used to induce double-stranded DNA breaks in the following cells: mouse iPSCs induced using lentivirus (lv-iPSCs) or chemically with CHR99021, Repsox and forskolin (ci-iPSCs) [21]; mouse ESCs; and mouse embryonic fibroblasts (MEFs) [22–26].

The experiments showed that lv-iPSCs are more likely than the other cell types to harbor genomic abnormalities, likely due to lower genomic fidelity of DNA damage repair. We also found greater genomic stability in ci-iPSCs than lv-iPSCs.

## Methods

### Cell lines and culture

The lv- and ci-iPSCs were derived from female transgenic OG2 mice carrying an *Oct4*-GFP transgene. Both types of iPSCs and ESCs were cultured in Dulbecco’s Modified Eagle Medium (DMEM; Gibco, Grand Island, NY, USA) supplemented with 15% fetal bovine serum (FBS; Gibco), 1% MEM non-essential amino acids (Gibco), 1% penicillin/streptomycin (Gibco), 2 mmol/L l-glutamine (Gibco), 1 × 10^3^ units/mL of mouse leukemia inhibitory factor (Millipore, Temecula, CA, USA) and 0.1 mmol/L 2-mercaptoethanol (Gibco) [27]. The medium was changed daily, and cells were passaged every 2 days using 0.25% trypsin (Thermo Fisher Scientific, Beijing, China) [28]. MEFs were cultured in DMEM supplemented with 15% FBS, 1% non-essential amino acids and 1% penicillin/streptomycin [29].

### Irradiation

Cells were passaged 1 day before γ-irradiation (4 Gy) with a cobalt irradiator (Thermo Fisher Scientific). After the irradiation, cells were immediately returned to the incubator, and cultured for 4 h prior to analyses as described below.

### Western blotting

To test the phosphorylation level of ATM, cells were lysed in ATM lysis buffer [20 mmol/L HEPES (pH 7.4), 150 mmol/L NaCl, 0.2% Tween-20, 1.5 mmol/L MgCl_2_, 1 mmol/L EGTA, 2 mmol/L dithiothreitol, 50 mmol/L NaF, 500 μmol/L NaVO_4_, 1 mmol/L phenylmethylsulfonyl fluoride, 0.1 μg/mL aprotinin and 0.1 µg/mL leupeptin], and centrifuged, as describe previously [30].

In assays of histone modification, cells were re-suspended in 1-mL triton extraction buffer (TEB) containing 0.5% Triton X-100 and 2 mmol/L PMSF, and then lysed on ice for 10 min. The lysates were centrifuged at 1500*g* for 10 min at 4 °C. The pellet was washed with 1.5-mL TEB, re-suspended in 0.2 mol/L HCl, and incubated at 4 °C overnight. Samples were centrifuged at 6500*g* for 10 min, after which 200-µL supernatant was transferred to a new tube, and neutralized with 20-µL 2 mol/L NaOH.

Samples were separated using SDS-PAGE and transferred to PVDF membranes (Millipore, Billerica, MA, USA). Blots were incubated with a primary antibody against one of the following proteins: phospho-ATM (1:1000; R&D Systems, Minneapolis, MN, USA), β-actin (1:3000; Beyotime Biotech, Beijing, China), H3 (1:30,000; Abcam, Cambridge, MA, USA) and H3K9me3 (1:3000; Abcam). Blots were washed three times with phosphate-buffered saline (PBS), and then incubated with a horseradish peroxidase-conjugated anti-mouse secondary antibody (1:3000; Gene Tex, San Diego, CA, USA) or anti-rabbit secondary antibody (1:3000; Abcam). Protein bands of interest were visualized using an Image Quant ECL system (GE Healthcare, Piscataway, NJ, USA).

### Immunofluorescence labeling of γ-H_2_AX foci

Cells were passaged onto slides, exposed 24 h later to 4 Gy of γ-irradiation, and incubated at 37 °C for 4 h. Cells were washed with PBS, fixed with 4% paraformaldehyde for 10 min at room temperature, washed again with PBS, permeabilized for 10 min using 0.05% Triton X-100 and 0.5% NP-40, and then washed three times (5 min each) in PBS. The cells were blocked for 1 h with 2% bovine serum albumin (BSA), and then incubated for 1 h at room temperature with a mouse anti-γH_2_AX antibody (1:500; Millipore, Temecula, CA, USA). Cells were washed three times with PBS containing 0.05% Tween 20, and then incubated with a goat anti-mouse secondary antibody (1:800; Abcam) for 1 h in the dark at room temperature. Cells were counterstained with 0.2 mg/mL 4′,6-diamidino-2-phenylindole (DAPI, 1:2000; Sigma, Shanghai, China). Confocal images were acquired and analyzed using a TCS SP5 (Leica) microscope equipped with an HCX PL 63 × 1.4 CS oil-immersion objective lens.

### DNA extraction

Three types of cells (lv-iPSCs, ci-iPSCs, ESCs) were digested with 0.25% trypsin and re-suspended in gelatin-coated dishes. After incubation at 37 °C for 15 min, supernatants were transferred to 15-mL centrifuge tubes, and cells were collected by centrifugation at 500*g* for 5 min at room temperature. DNA was extracted using a QIAamp DNA Mini Kit (Qiagen, Hilden, Germany).

### Whole-genome re-sequencing

Whole-genome DNA libraries suitable for sequencing using an Illumina sequencing platform were generated from 1-µg genomic DNA. The DNA was sheared to approximately 300–500 bp using a Covaris S220 instrument (Life Technologies, Carlsbad, CA, USA). A total of 2× 101-bp paired-end reads were produced using the HiSeq 2000 DNA Sequencer.

The sequencing data were mapped to a reference mouse genomic sequence (mm9) using the Burrows–Wheeler alignment tool algorithm [31]. Unique alignment reads were retained for later analysis. Using the untreated cells as a control, single-nucleotide variations (SNVs) were collected using the “mpileup” tool in SAMTools as well as the UnifiedGenotyper in the GATK module [32, 33]. Quality recalibration and local realignment were performed using GATK tools before variation calling was performed. The following criteria were applied for calling mutations using pairwise samples: (1) the minimum coverage of variant sites had to be greater than 20 and base quality greater than 15; (2) the frequency of mutant SNVs had to be 0 in control samples and 0.2 in irradiated samples; and (3) the variant sites had to be supported by at least two reads on the forward strand and two reads on the reverse strand.

### RNA sequencing

Total RNA was extracted from each cell line using TRIzol reagent and enriched for mRNA using oligo (dT) magnetic beads. Approximately 1-µg mRNA was fragmented and electrophoresed to isolate mRNA fragments (200–250 bases). These fragments were subjected to end repair, 3′ terminal adenylation and adapter ligation, followed by cDNA synthesis. The resulting cDNAs were gel-electrophoresed to isolate 250–300 bp fragments, and were sequenced using a HiSeq 2000 system (Illumina).

Sequencing reads were aligned to a reference sequence (GRCm37/mm9) using TopHat alignment software [34, 35]. Only uniquely aligned reads were used for transcript assembly, which was performed using Cufflinks software [36]. Read counts for each gene were calculated, and the expression levels of each gene were normalized using the “fragments per kilobase of exon model per million mapped” (FPKM) algorithm. Differentially expressed genes were filtered based on false discovery rate (FDR)-adjusted *P* < 0.05. The profile of differentially expressed genes was visualized and analyzed using the Bioconductor function “CummeRbund” in the R program [37]. Hierarchical clustering was performed using the “heatmap” package in R.

### Generation of iPSC- and ESC-derived mice

Two cell-stage ICR embryos were electrofused to produce tetraploid embryos, and 10–15 iPSCs and ESCs were subsequently injected into the reconstructed tetraploid blastocysts. Embryos were cultured for 1 day prior to transplantation into the uterus of pseudo-pregnant mice. Caesarean sections were performed at E19.5, and the pups were fostered by lactating ICR mothers [38].

### Comet assay

Mice derived from iPSCs or ESCs as well as C57 mice were treated with 4 Gy ionizing radiation. At 4 h later, bone marrow cells were isolated and re-suspended using PBS and concentrated by adding 150-μL molten 0.75% low-melting-point agarose. An aliquot of concentrated cells (60 μL) was then added to molten 0.8% normal-melting-point agarose on comet slides. The slides were incubated for 1–2 h at 4 °C with pre-chilled lysis buffer, stored in the dark at 4 °C for 20 min, then incubated with pre-chilled electrophoresis buffer (0.3 mol/L NaOH containing 0.5 mol/L EDTA, pH > 13.0). Gel electrophoresis was performed at 25 V for 20 min at 4 °C. Slides were incubated at 4 °C for 15 min with neutralization buffer (0.4 mol/L Tris, pH > 7.5), washed with 100% ethanol for 3–5 min and air-dried at room temperature. Diluted ethidium bromide (EB) solution (20–30 μL) was placed onto each dried agarose circle. Slides were then read at 100 cells/sample using a fluorescence microscope equipped with CASP DNA damage analysis software.

## Results

### Similar gene expression profile between lv-iPSCs and ESCs

**Fig. 1:**
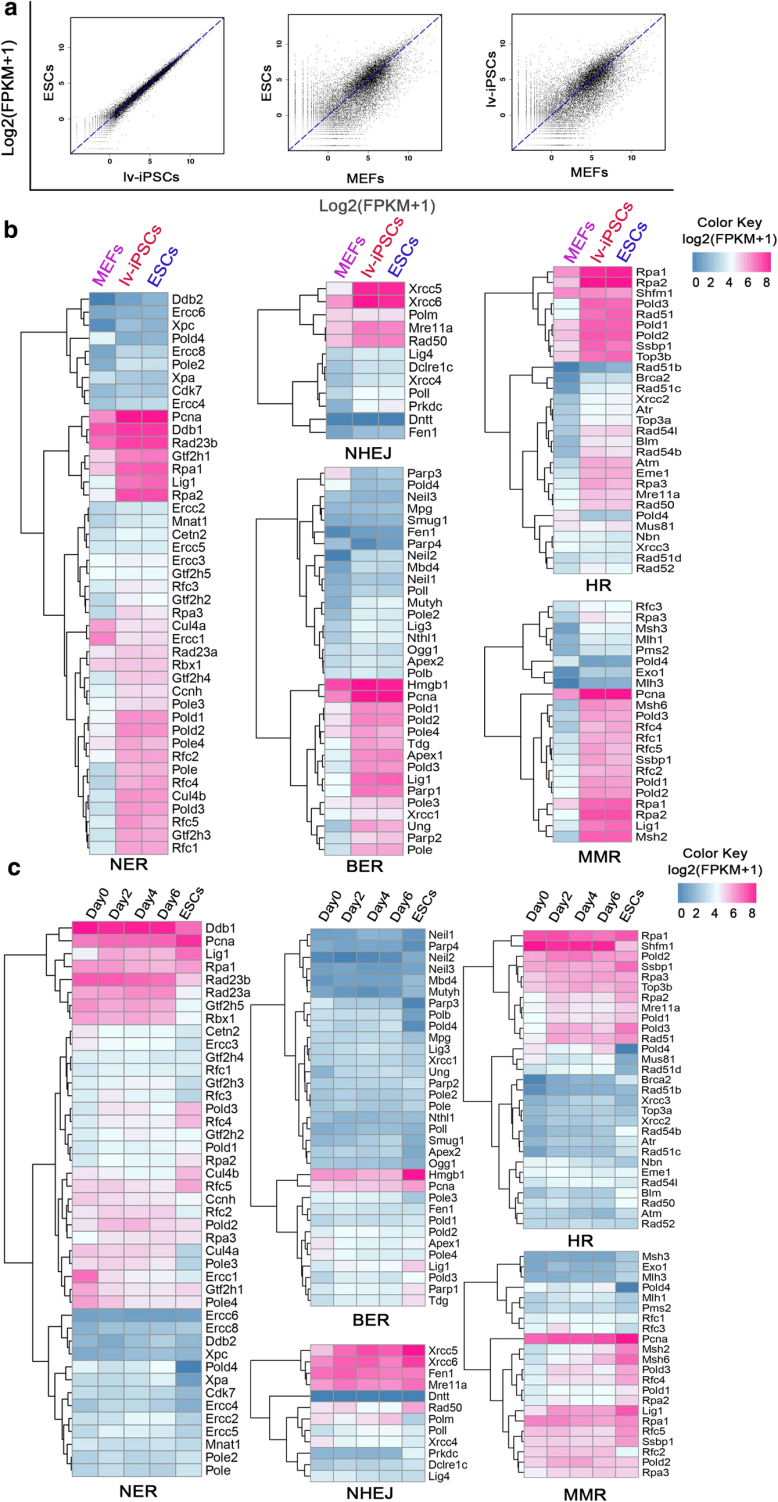
Gene expression profile of ESCs, lv-iPSCs and MEFs. **a** Scatter plots used to identify global trends in gene expression and differences among cell lines. **b** Heat maps showing the expression level of DNA damage repair-associated genes in the cell lines. Blue color indicates lowest expression; fuchsia, highest. **c** Re-analysis of the expression of DNA damage repair-associated genes during early reprogramming

RNA-seq analysis showed that the gene expression profile of lv-iPSCs was similar to that of ESCs but not to that of MEFs (Fig. 1a), indicating iPSC pluripotency. Since genomic stability depends on DNA damage repair, we analyzed expression of the genes involved in DNA damage repair pathways. No significant differences in the expression of such genes were found between lv-iPSCs and ESCs (Fig. 1b). We further analyzed the expression of DNA repair genes that were identified during early reprogramming of iPSCs in our previous report [39] and confirmed the up-regulation of those genes at early iPSC stages (Fig. 1c). These results suggest that DNA damage repair pathways can be reprogrammed at early iPSC stages and become similar to pathways in ESCs as reprogramming continues [39].

### More DNA mutations in lv-iPSCs than in other cell types after ionizing irradiation

**Fig. 2:**
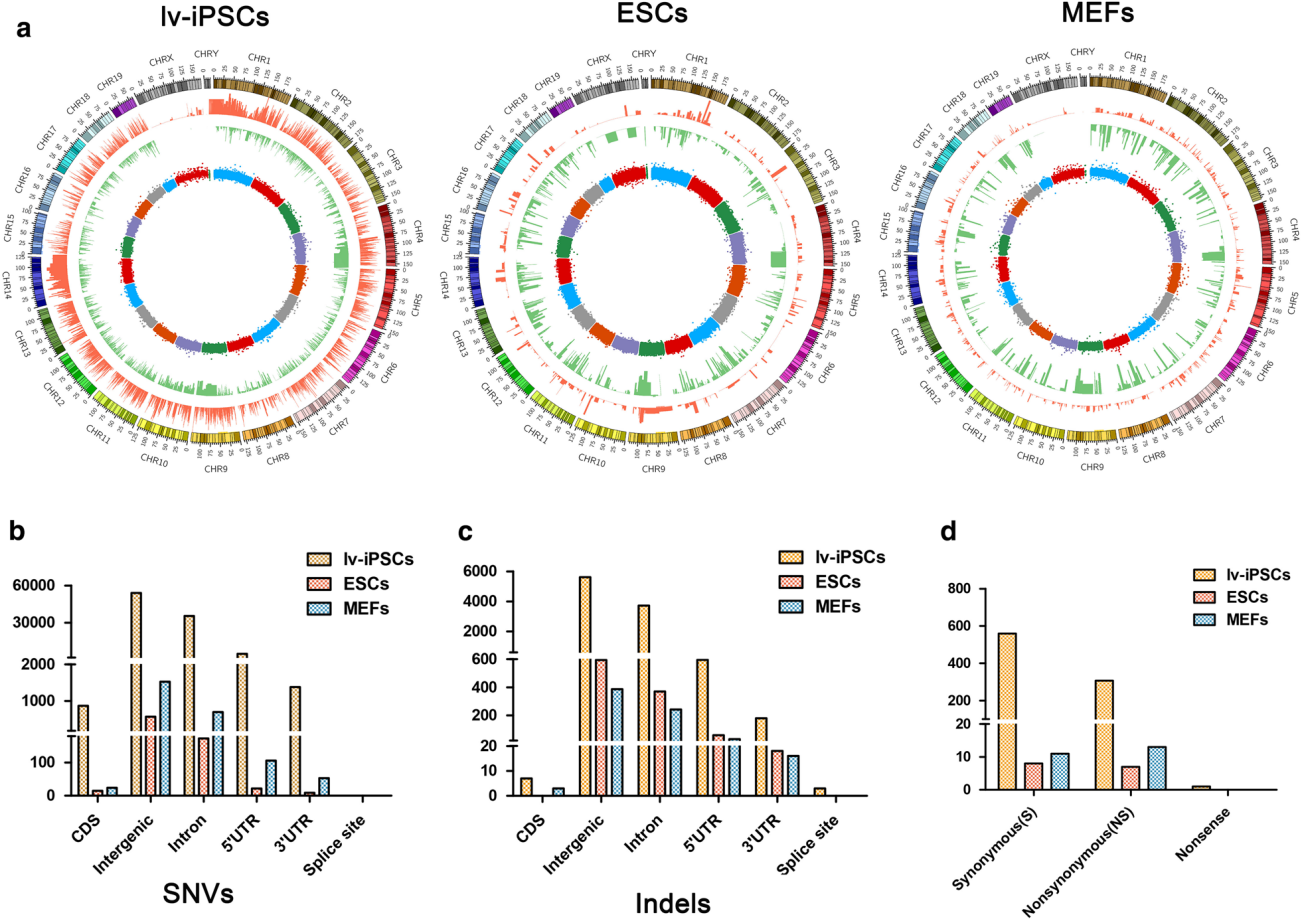
Genomic variation in each cell line after ionizing irradiation. **a** Circos plot showing genetic alterations in lv-iPSCs, ESCs and MEFs after irradiation, based on the corresponding untreated cells as the reference. CHR, chromosome. **b**, **c** Histograms showing the numbers of (**b**) single-nucleotide variations (SNVs) and (**c**) short insertions or deletions (indels) in each type of genomic region in each cell line. **d** Histogram of the number of SNVs in a coding region (CDS) in each cell line

We treated mouse lv-iPSCs, ESCs and MEFs with 4 Gy ionizing radiation to induce double-strand breaks. If not repaired properly, such breaks can result in genomic abnormalities, apoptosis and senescence [23, 26, 40]. Whole-genome DNA sequencing at 4 h after irradiation revealed more SNVs in lv-iPSCs than in the other cell types (Fig. 2a, Table 1), as well as more short indels (Fig. 2a, Table 2). MEFs showed a larger variety of copy number variations (CNVs) than the other cell types (Fig. 2a).

**Table 1:** Summary of sequencing results

Parameter	lv-iPSCs		ESCs		MEFs	
	IR−	IR+	IR−	IR+	IR−	IR+
Total nucleotides sequenced (Gb)	64.1	71.5	72.0	70.9	64.7	70.1
Genome coverage (fold)	20×	21×	22×	23×	20×	21×
Total number of reads	634,852,868	708,065,514	765,056,092	754,255,820	640,447,936	693,376,402

*ESCs* mouse embryonic stem cells, *IR* ionizing radiation, *lv-iPSCs* lentivirus induced iPS cells, *MEFs* mouse embryonic fibroblasts

**Table 2:** Summary of somatic indels in each cell line

	lv-iPSCs	ESCs	MEFs
Somatic indels	10,127	1041	679
CDS	7	0	3
Intergenic	5616	594	387
Intron	3725	370	242
5′ UTR	596	59	31
3′ UTR	180	18	16
Splice site	3	0	0

*CDS* coding sequence, *indel* insertion or deletion, *UTR* untranslated region

A larger number of SNVs and indels occurred in coding regions, intergenic regions, introns, 5′ untranslated regions (UTRs) and 3′ UTRs of lv-iPSCs than in other cell types (Fig. 2b, c). Irradiation was associated with the appearance of many more synonymous point mutations in coding regions in lv-iPSCs (559) than in ESCs (8) or MEFs (11) (Fig. 2d, Table 3). Similarly, many more non-synonymous point mutations in coding regions were found in lv-iPSCs (307) than in ESCs (7) or MEFs (13) (Fig. 2d, Tables 3, 4, 5, 6).

**Table 3:** Summary of somatic mutations in each cell line

	lv-iPSCs	ESCs	MEFs
Somatic mutation	92,027	789	2403
CDS	867	15	24
Intergenic	54,086	570	1526
Intron	35,567	173	694
5′ UTR	5128	22	106
3′ UTR	1379	9	53
Splice site	0	0	0
CDS	867	15	24
Synonymous	559	8	11
Nonsynonymous	307	7	13
Nonsense	1	0	0

*CDS* coding sequence, *indel* insertion or deletion, *UTR* untranslated region

**Table 4:** Frequencies of coding SNVs in ESCs exposed to ionizing radiation

#	Locus	Gene	Mutation	Amino acid change	Freq. IR− (%)	Freq. IR+ (%)
1	Chr1:46244148	Dnahc7b	T−> C	V−>A	0.0	28.57
2	Chr1:172987924	Fcgr3	A−>C	S−>A	0.0	30
3	Chr2:180330052	Ogfr	A−>G	E−>G	0.0	22.06
4	Chr8:22465823	Defa-rs1	C−>G	R−>P	0.0	20
5	Chr10:80640826	Eef2	G−>A	E−>K	0.0	30.11
6	Chr15:77465439	Apol11b	C−>A	K−>N	0.0	20
7	chrX:131293286	Armcx3	G−>A	C−>Y	0.0	21.62

*IR−* control cells not irradiated, *IR+* irradiated cells, *SNV* single-nucleotide variants

**Table 5:** Frequencies of coding SNVs in MEFs exposed to ionizing radiation

#	Locus	Gene	Mutation	Amino acid change	Freq. IR− (%)	Freq. IR+ (%)
1	Chr1:174406441	Slamf9	T−>C	M−>T	0.0	23.68
2	Chr2:10014034	Kin	G−>A	E−>K	0.0	20.00
3	Chr4:120619977	Zfp69	A−>G	S−>P	0.0	20.00
4	Chr4:146162835	Zfp600	T−>A	I−>K	0.0	20.00
5	Chr5:72655729	Atp10d	C−>G	P−>R	0.0	27.78
6	Chr7:25371714	Zfp575	G−>A	A−>V	0.0	25.00
7	Chr7:31658366	Cd22	C−>T	R−>Q	0.0	30.56
8	Chr7:48249435	4930433I11Rik	A−>T	D−>V	0.0	23.08
9	Chr7:48249440	4930433I11Rik	G−>C	A−>P	0.0	24.00
10	Chr8:93611040	Rbl2	G−>C	R−>T	0.0	20.83
11	Chr14:52076074	Vmn2r89	C−>T	A−>V	0.0	20.00
12	Chr18:67019622	Mc4r	C−>T	G−>S	0.0	22.50
13	Chr18:70668975	Poli	C−>T	G−>R	0.0	29.03

*IR−* control cells not irradiated, *IR+* irradiated cells, *SNV* single-nucleotide variants

**Table 6:** Frequencies of coding SNVs in lv-iPSCs exposed to ionizing radiation

#	Locus	Gene	Mutation	Amino acid change	Freq. IR− (%)	Freq. IR+ (%)
1	chr1:30861639	Phf3	C−>G	E−>Q	0.00	20.93
2	chr1:60166069	Carf	C−>T	R−>W	0.00	42.86
3	chr1:92665043	Col6a3	C−>G	E−>D	0.00	22.73
4	chr1:108649819	Kdsr	G−>T	D−>E	0.00	27.59
5	chr1:152550404	Hmcn1	C−>T	V−>I	0.00	25.00
6	chr1:166275528	Nme7	G−>A	G−>S	0.00	32.26
7	chr1:171863885	1700084C01Rik	G−>A	G−>S	0.00	25.00
8	chr1:175866740	Ifi203	G−>A	T−>M	0.00	28.57
9	chr1:186630980	Mosc1	G−>C	D−>E	0.00	23.08
10	chr1:186740013	Mark1	T−>A	E−>D	0.00	32.14
11	chr2:10112008	Itih5	T−>C	S−>P	0.00	42.31
12	chr2:31655794	Abl1	A−>G	S−>G	0.00	25.00
13	chr2:31656413	Abl1	A−>C	N−>T	0.00	24.24
14	chr2:34634942	Rabepk	T−>C	K−>E	0.00	44.44
15	chr2:34858984	Hc	C−>T	S−>N	0.00	20.51
16	chr2:79182476	Cerkl	C−>T	A−>T	0.00	24.00
17	chr2:86000090	Olfr1042	T−>C	T−>A	0.00	42.86
18	chr2:86154881	Olfr1053	A−>C	I−>M	0.00	42.86
19	chr2:86828637	Olfr1101	T−>G	Q−>P	0.00	41.67
20	chr2:87149857	Olfr1118	A−>G	K−>E	0.00	40.74
21	chr2:89033480	Olfr1226	G−>A	S−>F	0.00	57.14
22	chr2:90749357	Kbtbd4	A−>G	I−>V	0.00	36.36
23	chr2:90894429	Psmc3	A−>G	T−>A	0.00	23.53
24	chr2:91757766	Ambra1	G−>A	R−>Q	0.00	60.71
25	chr2:92815310	Prdm11	G−>A	S−>L	0.00	43.33
26	chr2:119346136	Exd1	T−>A	H−>L	0.00	35.00
27	chr2:119577973	Ltk	C−>T	G−>E	0.00	34.38
28	chr2:120104674	Pla2g4d	G−>A	P−>L	0.00	20.00
29	chr2:120265164	Ganc	C−>G	I−>M	0.00	40.00
30	chr2:120357660	Zfp106	G−>T	Q−>K	0.00	42.31
31	chr2:126412071	Slc27a2	G−>T	A−>S	0.00	24.00
32	chr2:127182455	Astl	C−>T	P−>L	0.00	30.56
33	chr2:127267842	Fahd2a	C−>A	G−>W	0.00	21.74
34	chr2:146172498	Ralgapa2	C−>T	V−>I	0.00	25.00
35	chr2:150299134	Zfp345	A−>T	L−>Q	0.00	24.00
36	chr2:153757199	Bpifb3	G−>A	M−>I	0.00	33.33
37	chr2:157822874	Tti1	T−>C	K−>R	0.00	20.59
38	chr2:157832871	Tti1	C−>T	S−>N	0.00	21.74
39	chr2:165177990	Zfp663	C−>T	R−>Q	0.00	21.74
40	chr2:165880571	Ncoa3	G−>A	S−>N	0.00	43.48
41	chr2:174471852	Zfp831	T−>C	S−>P	0.00	25.93
42	chr3:19570978	Trim55	G−>A	G−>S	0.00	29.17
43	chr3:20127155	Cpa3	T−>A	K−>I	0.00	36.36
44	chr3:65861245	Veph1	A−>G	S−>P	0.00	25.00
45	chr3:88240586	Sema4a	G−>A	A−>V	0.00	29.41
46	chr3:94167523	C2cd4d	G−>C	R−>P	0.00	22.22
47	chr3:96096266	Fcgr1	G−>A	P−>S	0.00	20.00
48	chr3:97414088	Chd1l	T−>C	S−>G	0.00	42.86
49	chr3:105789443	Ovgp1	C−>T	T−>I	0.00	21.74
50	chr3:116192199	Rtcd1	C−>T	V−>I	0.00	27.27
51	chr3:118377426	Dpyd	G−>A	S−>N	0.00	41.67
52	chr3:137770265	Mttp	T−>A	T−>S	0.00	26.09
53	chr3:142271248	Gbp1	G−>A	E−>K	0.00	30.95
54	chr4:57660898	Palm2	G−>A	V−>I	0.00	40.00
55	chr4:106415886	Fam151a	G−>A	R−>Q	0.00	51.85
56	chr4:116265516	Gpbp1l1	T−>A	S−>T	0.00	25.00
57	chr4:118154980	Tie1	T−>C	D−>G	0.00	30.30
58	chr4:119804939	Hivep3	T−>C	L−>P	0.00	25.00
59	chr4:120620061	Zfp69	T−>C	T−>A	0.00	40.00
60	chr4:120620067	Zfp69	T−>G	T−>P	0.00	37.04
61	chr4:136193988	Lactbl1	A−>G	S−>G	0.00	33.33
62	chr4:141674086	Kazn	C−>T	A−>T	0.00	20.00
63	chr4:147839151	Mtor	C−>T	R−>C	0.00	25.71
64	chr5:23825901	Kcnh2	T−>G	T−>P	0.00	34.78
65	chr5:23905831	Abcb8	T−>C	W−>R	0.00	20.00
66	chr5:64289838	0610040J01Rik	T−>A	L−>Q	0.00	24.00
67	chr5:90672580	Ankrd17	A−>G	*−>Q	0.00	28.89
68	chr5:109231028	Vmn2r8	T−>C	E−>G	0.00	20.83
69	chr5:122789758	Rad9b	A−>G	L−>S	0.00	37.93
70	chr5:138473740	Smok3a	A−>G	Q−>R	0.00	32.00
71	chr5:142948192	C330006K01Rik	G−>A	G−>R	0.00	22.86
72	chr5:146996767	1700001J03Rik	C−>T	R−>H	0.00	30.56
73	chr6:67242225	Il12rb2	T−>C	Y−>C	0.00	21.21
74	chr6:67423944	Il23r	T−>C	T−>A	0.00	26.09
75	chr6:72529697	Elmod3	T−>C	H−>R	0.00	25.00
76	chr6:123355291	Vmn2r20	G−>A	A−>V	0.00	24.00
77	chr6:124820464	Cd4	G−>A	P−>S	0.00	30.77
78	chr6:128334974	4933413G19Rik	G−>A	G−>R	0.00	23.53
79	chr6:129369539	Clec9a	A−>G	N−>S	0.00	40.91
80	chr6:132907129	Tas2r131	C−>T	R−>Q	0.00	26.09
81	chr6:141942744	Gm6614	C−>T	D−>N	0.00	32.14
82	chr6:142186044	Slco1a5	G−>A	S−>L	0.00	25.00
83	chr6:142186083	Slco1a5	G−>T	P−>H	0.00	27.27
84	chr6:142201619	Slco1a5	T−>G	D−>A	0.00	35.29
85	chr6:142251831	Iapp	G−>C	S−>T	0.00	26.92
86	chr7:3794286	Pira2	A−>G	S−>P	0.00	26.09
87	chr7:7278011	Vmn2r30	T−>A	N−>I	0.00	22.22
88	chr7:10859910	Vmn1r66	G−>A	H−>Y	0.00	38.10
89	chr7:11333654	Vmn1r71	G−>A	T−>I	0.00	50.00
90	chr7:12738597	Vmn1r78	T−>C	F−>S	0.00	20.00
91	chr7:17743709	Ceacam3	C−>G	L−>V	0.00	20.00
92	chr7:17743712	Ceacam3	A−>C	I−>L	0.00	22.50
93	chr7:18337478	Ceacam5	G−>A	R−>Q	0.00	22.73
94	chr7:18662759	Ceacam12	G−>C	G−>A	0.00	26.09
95	chr7:19672969	Dmpk	G−>A	A−>T	0.00	33.33
96	chr7:26134047	Megf8	C−>T	H−>Y	0.00	20.45
97	chr7:26261696	Ceacam1	C−>G	A−>P	0.00	20.00
98	chr7:29779122	Map4k1	T−>C	C−>R	0.00	21.05
99	chr7:31370138	Wbp7	C−>A	A−>S	0.00	31.82
100	chr7:31374957	Zbtb32	C−>T	A−>T	0.00	30.77
101	chr7:31391976	Upk1a	G−>A	T−>I	0.00	31.03
102	chr7:31696435	Mag	C−>T	V−>I	0.00	40.00
103	chr7:48299575	Gm4884	G−>T	A−>S	0.00	21.28
104	chr7:48299666	Gm4884	A−>C	H−>P	0.00	22.22
105	chr7:51608348	Shank1	G−>A	G−>S	0.00	20.00
106	chr7:54720024	Mrgpra2b	T−>A	H−>L	0.00	42.42
107	chr7:55424237	Mrgprb5	A−>G	I−>T	0.00	30.43
108	chr7:55424238	Mrgprb5	T−>A	I−>F	0.00	29.17
109	chr7:86855301	Kif7	C−>T	R−>H	0.00	42.42
110	chr7:89455314	Sh3gl3	T−>G	S−>A	0.00	40.00
111	chr7:108978612	Inppl1	G−>A	H−>Y	0.00	35.29
112	chr7:109584206	Stim1	T−>A	L−>H	0.00	24.14
113	chr7:109762976	Olfr553	G−>T	L−>M	0.00	23.26
114	chr7:109832588	Trim68	T−>C	I−>V	0.00	32.00
115	chr7:109862636	Olfr33	A−>G	F−>S	0.00	27.78
116	chr7:109872941	Olfr559	A−>T	I−>N	0.00	23.53
117	chr7:110121916	Olfr577	C−>T	A−>T	0.00	34.48
118	chr7:110234973	Olfr584	T−>C	F−>L	0.00	22.58
119	chr7:110336027	Olfr592	A−>G	H−>R	0.00	33.33
120	chr7:110399181	Dub2a	C−>G	E−>Q	0.00	42.31
121	chr7:110566860	Usp17l5	C−>A	P−>T	0.00	25.00
122	chr7:111161069	Olfr639	A−>G	I−>T	0.00	25.71
123	chr7:111161070	Olfr639	T−>C	I−>V	0.00	27.78
124	chr7:111297423	Ubqlnl	C−>G	Q−>H	0.00	23.33
125	chr7:111298754	Ubqlnl	T−>G	T−>P	0.00	31.58
126	chr7:111302579	E030002O03Rik	A−>G	V−>A	0.00	20.00
127	chr7:111560797	Trim30a	G−>C	T−>S	0.00	33.33
128	chr7:111793632	Olfr658	T−>C	T−>A	0.00	25.00
129	chr7:112009277	Dub1	C−>T	R−>C	0.00	35.00
130	chr7:112041695	Olfr666	G−>A	A−>V	0.00	29.03
131	chr7:112123974	Olfr671	C−>A	S−>I	0.00	20.00
132	chr7:112462829	Olfr689	G−>T	A−>S	0.00	21.95
133	chr7:112708033	Apbb1	G−>A	S−>F	0.00	26.32
134	chr7:114029870	Olfr706	G−>A	L−>F	0.00	23.08
135	chr7:114218082	Olfr714	G−>A	V−>I	0.00	21.43
136	chr7:115302565	Olfr485	C−>T	G−>E	0.00	28.00
137	chr7:115399125	Olfr488	T−>C	K−>E	0.00	25.00
138	chr7:115968770	Olfr514	T−>C	T−>A	0.00	25.00
139	chr7:116859980	BC051019	G−>A	T−>I	0.00	26.09
140	chr7:135022359	Zfp646	C−>G	L−>V	0.00	23.68
141	chr7:135024297	Zfp646	G−>A	E−>K	0.00	20.83
142	chr7:135026037	Zfp646	A−>G	S−>G	0.00	28.89
143	chr8:4213992	BC068157	G−>A	P−>L	0.00	52.38
144	chr8:80770162	Ttc29	C−>T	P−>L	0.00	22.86
145	chr8:86691455	4930432K21Rik	C−>A	P−>T	0.00	37.50
146	chr8:112256157	Atxn1l	C−>G	V−>L	0.00	21.88
147	chr9:21085574	Kri1	T−>C	K−>E	0.00	35.71
148	chr9:21733911	Ccdc159	G−>T	S−>I	0.00	51.72
149	chr9:22004013	Zfp872	C−>T	L−>F	0.00	34.88
150	chr9:22005064	Zfp872	G−>A	G−>E	0.00	34.15
151	chr9:22005066	Zfp872	T−>C	*−>R	0.00	32.50
152	chr9:22058381	Zfp599	C−>T	M−>I	0.00	34.62
153	chr9:35646988	9230110F15Rik	A−>G	V−>A	0.00	22.22
154	chr9:36671150	Fez1	A−>C	E−>D	0.00	34.62
155	chr9:37869528	Olfr885	G−>A	V−>M	0.00	27.27
156	chr9:41932183	Sorl1	C−>T	S−>N	0.00	32.35
157	chr9:44073278	Nlrx1	A−>C	F−>V	0.00	25.00
158	chr9:45557815	Dscaml1	G−>C	K−>N	0.00	42.86
159	chr9:50490277	Dixdc1	C−>T	R−>Q	0.00	21.74
160	chr9:55821819	Rfpl3s	G−>A	T−>M	0.00	29.03
161	chr9:58347114	6030419C18Rik	G−>A	A−>T	0.00	24.14
162	chr9:120873710	Ulk4	T−>C	I−>V	0.00	23.68
163	chr10:18244674	Nhsl1	G−>C	C−>S	0.00	21.88
164	chr10:18722769	Tnfaip3	A−>G	L−>P	0.00	28.00
165	chr10:51201543	Gp49a	C−>T	P−>S	0.00	22.22
166	chr10:51201551	Gp49a	T−>A	H−>Q	0.00	20.00
167	chr10:51203657	Gp49a	T−>C	Y−>H	0.00	27.50
168	chr10:51203677	Gp49a	T−>G	N−>K	0.00	44.74
169	chr10:53257912	Mcm9	A−>T	S−>T	0.00	37.50
170	chr10:61892173	Supv3l1	C−>T	D−>N	0.00	31.43
171	chr10:62301871	Tet1	T−>C	E−>G	0.00	27.59
172	chr10:62534718	Pbld1	G−>T	G−>V	0.00	26.67
173	chr10:62534721	Pbld1	G−>A	G−>E	0.00	26.67
174	chr10:69997479	Fam13c	T−>G	S−>A	0.00	23.68
175	chr10:82654374	Chst11	G−>A	G−>S	0.00	23.08
176	chr10:85391311	Ascl4	G−>C	G−>R	0.00	28.57
177	chr10:99909744	Tmtc3	C−>T	R−>K	0.00	30.00
178	chr10:99914062	Tmtc3	C−>T	R−>K	0.00	44.83
179	chr10:100031465	Cep290	A−>C	M−>L	0.00	47.62
180	chr10:128448679	Olfr763	T−>A	C−>S	0.00	20.00
181	chr11:5587351	Ankrd36	G−>A	V−>I	0.00	33.33
182	chr11:5587391	Ankrd36	A−>T	K−>I	0.00	22.22
183	chr11:6501551	Nacad	G−>A	P−>S	0.00	39.39
184	chr11:23264045	Usp34	A−>T	E−>D	0.00	28.57
185	chr11:29429943	Mtif2	A−>G	Q−>R	0.00	25.93
186	chr11:29607190	Rtn4	G−>C	S−>T	0.00	25.00
187	chr11:29607841	Rtn4	C−>T	A−>V	0.00	31.25
188	chr11:29646793	Eml6	G−>C	L−>V	0.00	37.14
189	chr11:32184064	Hba-a1	G−>C	G−>A	0.00	28.00
190	chr11:35622812	Rars	G−>T	A−>E	0.00	22.22
191	chr11:48988354	Btnl9	T−>C	Q−>R	0.00	39.13
192	chr11:52216575	9530068E07Rik	C−>T	A−>V	0.00	34.38
193	chr11:62078872	Adora2b	G−>A	R−>H	0.00	25.71
194	chr11:67688822	Usp43	T−>C	M−>V	0.00	24.00
195	chr11:69010998	Alox8	A−>G	V−>A	0.00	21.43
196	chr11:70584746	Zfp3	C−>A	P−>T	0.00	20.83
197	chr11:70995613	Nlrp1b	A−>T	F−>Y	0.00	21.74
198	chr11:70995614	Nlrp1b	A−>G	F−>L	0.00	23.81
199	chr11:70995616	Nlrp1b	A−>C	I−>R	0.00	22.73
200	chr11:72984698	P2rx5	C−>T	A−>V	0.00	25.00
201	chr11:96214596	Hoxb2	G−>A	E−>K	0.00	61.90
202	chr11:96772447	Cdk5rap3	C−>T	V−>I	0.00	47.50
203	chr11:101045277	Cntnap1	C−>T	S−>L	0.00	31.25
204	chr11:102935952	Plcd3	A−>T	D−>E	0.00	23.81
205	chr11:106174196	Cd79b	T−>C	M−>V	0.00	22.22
206	chr11:120146302	Bahcc1	C−>G	T−>S	0.00	21.43
207	chr12:18521595	5730507C01Rik	A−>T	N−>Y	0.00	20.00
208	chr12:21271015	Asap2	C−>T	T−>I	0.00	21.43
209	chr12:21379212	Adam17	A−>C	S−>A	0.00	20.69
210	chr12:25723341	Kidins220	G−>A	G−>S	0.00	31.82
211	chr12:32005994	Lamb1	T−>C	V−>A	0.00	25.00
212	chr12:65573550	Fscb	A−>G	S−>P	0.00	25.00
213	chr12:77031626	Syne2	G−>A	R−>K	0.00	20.00
214	chr12:77088037	Syne2	A−>G	H−>R	0.00	37.93
215	chr12:77701313	Spnb1	G−>C	D−>E	0.00	27.08
216	chr12:77713010	Spnb1	A−>T	M−>K	0.00	26.47
217	chr12:80369378	Zfyve26	T−>C	Q−>R	0.00	28.00
218	chr12:85333734	Acot2	A−>G	T−>A	0.00	22.86
219	chr12:88947186	Oog1	G−>A	E−>K	0.00	20.45
220	chr12:111906810	1700001K19Rik	T−>G	Q−>P	0.00	29.82
221	chr13:6564252	Pitrm1	C−>A	T−>K	0.00	32.14
222	chr13:6604968	Pfkp	C−>T	V−>M	0.00	34.48
223	chr13:8886000	Idi1	T−>C	S−>P	0.00	22.58
224	chr13:8958551	Idi2	A−>G	E−>G	0.00	27.27
225	chr13:9150373	Larp4b	T−>C	L−>S	0.00	34.48
226	chr13:9688439	Zmynd11	C−>T	S−>N	0.00	33.33
227	chr13:14097474	Tbce	G−>A	A−>V	0.00	22.73
228	chr13:23126330	Vmn1r214	G−>A	E−>K	0.00	42.86
229	chr13:23126981	Vmn1r214	C−>A	Q−>K	0.00	40.91
230	chr13:23309404	Vmn1r221	C−>A	L−>I	0.00	30.77
231	chr13:23309956	Vmn1r221	C−>G	L−>V	0.00	25.93
232	chr13:23579753	Btn2a2	T−>A	I−>L	0.00	34.62
233	chr13:23647236	Hist1h1d	C−>T	T−>I	0.00	37.50
234	chr13:23855668	Hist1h1a	C−>T	A−>V	0.00	29.63
235	chr13:25085054	Mrs2	T−>C	T−>A	0.00	21.74
236	chr13:40238189	Ofcc1	G−>A	P−>S	0.00	20.83
237	chr13:58445640	Kif27	T−>A	I−>L	0.00	23.81
238	chr13:70874611	Adamts16	C−>T	G−>S	0.00	24.14
239	chr13:70877487	Adamts16	G−>C	Q−>E	0.00	20.59
240	chr13:81583863	Gpr98	C−>T	E−>K	0.00	34.48
241	chr13:96284081	F2rl1	C−>T	V−>I	0.00	25.00
242	chr13:98737291	Rgnef	G−>C	A−>G	0.00	33.33
243	chr14:45342600	Gm8267	A−>T	M−>K	0.00	28.00
244	chr14:50393514	3632451O06Rik	A−>G	V−>A	0.00	24.44
245	chr14:51135131	Olfr742	A−>G	N−>S	0.00	25.81
246	chr14:55283622	Acin1	T−>C	K−>E	0.00	43.18
247	chr14:70175997	Tnfrsf10b	C−>G	P−>A	0.00	27.78
248	chr14:70176001	Tnfrsf10b	T−>C	V−>A	0.00	25.00
249	chr14:78484964	AU021034	A−>G	C−>R	0.00	22.22
250	chr15:41697429	Abra	G−>C	L−>V	0.00	24.00
251	chr15:41701040	Abra	G−>C	L−>V	0.00	27.27
252	chr15:54965030	Deptor	A−>T	M−>L	0.00	25.00
253	chr15:66523859	Tg	G−>A	V−>I	0.00	20.00
254	chr15:75937421	Eppk1	C−>T	V−>I	0.00	25.00
255	chr15:76539950	Recql4	A−>G	L−>P	0.00	20.00
256	chr15:95455328	Dbx2	C−>T	V−>M	0.00	25.00
257	chr16:32756226	Muc4	C−>G	Q−>E	0.00	24.32
258	chr16:32779211	Muc4	C−>A	Q−>K	0.00	23.33
259	chr16:45577986	Slc9a10	C−>T	A−>V	0.00	20.00
260	chr16:56668453	Abi3bp	C−>A	P−>Q	0.00	22.22
261	chr16:58872574	Olfr176	C−>G	S−>T	0.00	20.59
262	chr17:6009735	Synj2	T−>A	F−>L	0.00	25.81
263	chr17:6037828	Synj2	C−>G	H−>D	0.00	25.00
264	chr17:7530924	Tcp10a	C−>T	P−>S	0.00	29.03
265	chr17:24111200	Prss30	A−>C	D−>E	0.00	21.05
266	chr17:24583771	E4f1	C−>T	S−>N	0.00	27.27
267	chr17:28021909	Uhrf1bp1	G−>A	G−>D	0.00	22.50
268	chr17:31365125	Ubash3a	C−>T	P−>S	0.00	25.81
269	chr17:31392140	Rsph1	G−>T	P−>Q	0.00	25.71
270	chr17:31398701	Rsph1	G−>A	T−>M	0.00	30.30
271	chr17:31754132	Cbs	T−>C	D−>G	0.00	29.03
272	chr17:32758655	Gm9705	G−>A	V−>M	0.00	20.00
273	chr17:33158753	Zfp763	C−>T	A−>T	0.00	22.86
274	chr17:33472542	Zfp81	A−>G	M−>T	0.00	27.59
275	chr17:34087468	B3galt4	T−>C	N−>S	0.00	28.21
276	chr17:34338203	Psmb8	G−>T	A−>S	0.00	28.57
277	chr17:34870392	C4b	C−>T	R−>Q	0.00	22.58
278	chr17:34974426	Dom3z	T−>C	L−>S	0.00	50.00
279	chr17:35267082	Apom	G−>T	Q−>K	0.00	43.33
280	chr17:35457904	H2-Q1	C−>T	P−>L	0.00	32.50
281	chr17:36168621	H2-T23	C−>G	R−>T	0.00	28.57
282	chr17:36218438	H2-Bl	T−>C	H−>R	0.00	27.03
283	chr17:36254622	H2-T10	G−>A	P−>S	0.00	33.33
284	chr17:36323554	H2-T3	T−>G	M−>L	0.00	21.67
285	chr17:43615815	Mep1a	T−>C	T−>A	0.00	25.00
286	chr17:43615911	Mep1a	T−>C	T−>A	0.00	30.00
287	chr17:43822205	Cyp39a1	G−>A	G−>R	0.00	28.57
288	chr17:46161537	Vegfa	G−>A	P−>L	0.00	37.04
289	chr17:46550212	Zfp318	A−>G	E−>G	0.00	29.03
290	chr17:46635998	BC048355	A−>C	K−>N	0.00	31.82
291	chr17:46893214	Ptcra	G−>T	R−>S	0.00	37.14
292	chr17:72047254	Fam179a	T−>G	F−>C	0.00	37.50
293	chr17:80734673	Arhgef33	G−>A	A−>T	0.00	26.67
294	chr17:88958139	Klraq1	T−>C	M−>T	0.00	29.03
295	chr17:89110812	Gtf2a1l	G−>A	R−>Q	0.00	31.03
296	chr17:89153211	Lhcgr	T−>C	T−>A	0.00	57.58
297	chr18:37907724	Pcdhga10	C−>A	H−>N	0.00	26.09
298	chr18:38132920	Arap3	G−>A	A−>V	0.00	44.83
299	chr18:60977711	Tcof1	C−>A	A−>S	0.00	56.00
300	chr18:60992401	Tcof1	C−>A	A−>S	0.00	46.43
301	chr18:65901869	5330437I02Rik	T−>C	F−>L	0.00	20.59
302	chr18:80326155	Adnp2	A−>G	F−>L	0.00	26.83
303	chr18:80389581	Rbfa	C−>T	A−>T	0.00	32.35
304	chr19:10751147	Pga5	C−>G	V−>L	0.00	45.83
305	chr19:11038598	Ms4a10	C−>T	V−>I	0.00	20.69
306	chr19:18912582	Trpm6	A−>G	M−>V	0.00	21.88
307	chr19:25696788	Dmrt3	C−>T	T−>M	0.00	29.63
308	chr7:111207208	Olfr643	G−>A	R−>*	0.00	29.41

*IR−* control cells not irradiated, *IR+* irradiated cells, *SNV* single-nucleotide variants

### Similar gene expression profile in lv-iPSCs with or without ionizing radiation

**Fig. 3:**
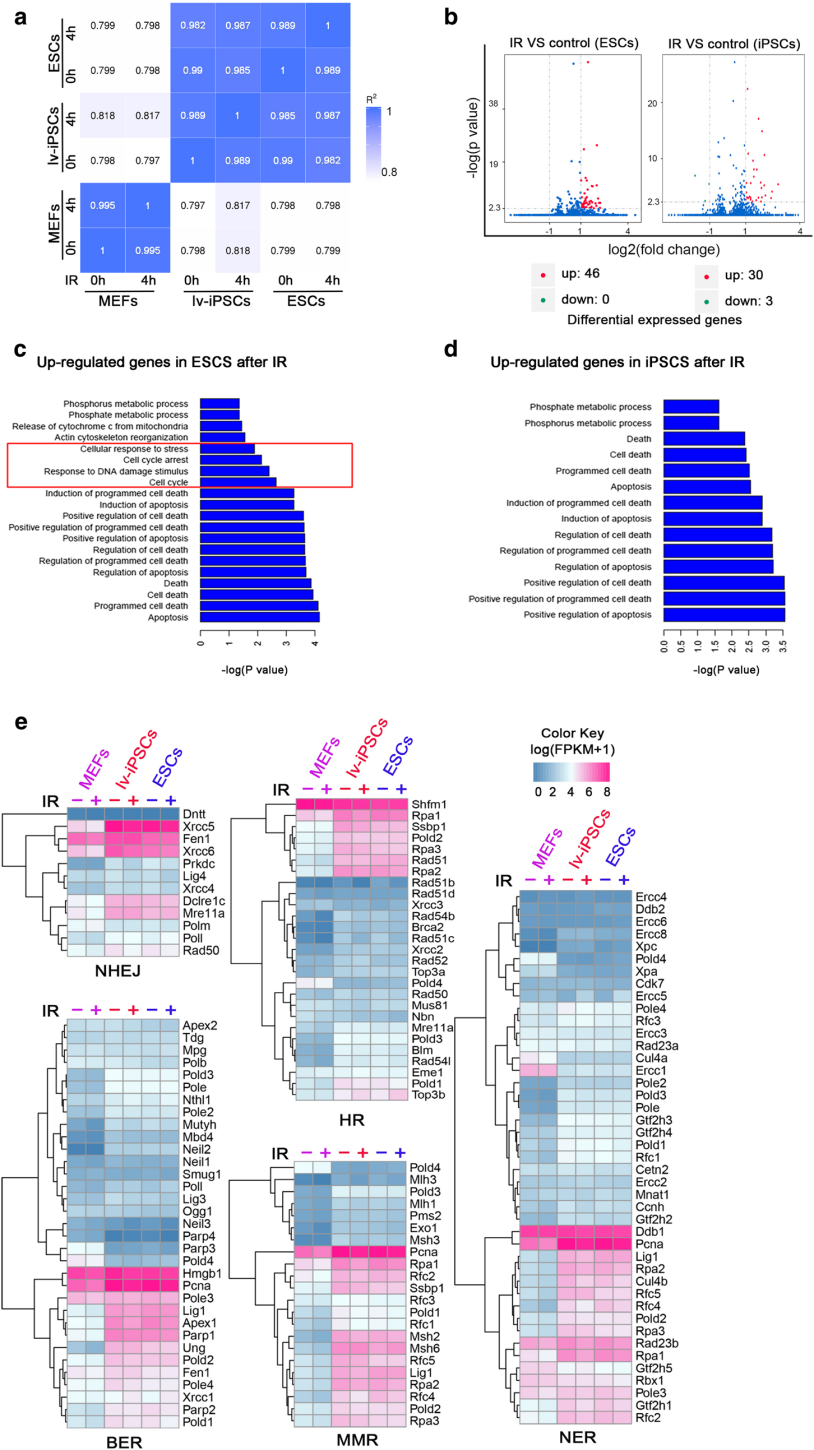
Gene expression levels in cells exposed or not to ionizing radiation (IR) for the indicated periods. **a** Heatmap showing Pearson’s correlation coefficients relating expression levels between irradiated and non-irradiated cells. **b** Volcano plots of genes expressed in irradiated and non-irradiated cells, showing genes significantly up-regulated (red dots) or down-regulated (green dots) in irradiated cells. Differentially expressed genes were filtered based on FDR < 0.05. **c**, **d** Histograms of gene ontology classifications of differentially expressed genes in irradiated (**c**) ESCs and (**d**) iPSCs. **e** Heat maps showing the expression level of DNA damage repair-associated genes in irradiated (+) and non-irradiated (−) cells. Blue indicates lowest expression; fuchsia, highest. *BER* base excision repair, *HR*, homologous recombination, *MMR* mismatch repair, *NER* nucleotide excision repair, *NHEJ* non-homologous end joining

To determine whether ionizing radiation alters the expression of certain genes in lv-iPSCs that may help explain the high mutation rate, RNA-seq analysis was conducted in irradiated versus control cells. The results indicated a similar gene expression profile with or without radiation (Fig. 3a). In fact, irradiation appeared to up-regulate only 46 genes in ESCs and 30 genes in lv-iPSCs (Fig. 3b). In contrast to the genes in lv-iPSCs that radiation up-regulated, majority of the genes up-regulated in ESCs is implicated in cellular response to stress and cell cycle processes (Fig. 3c, d).

Expression levels of genes involved in DNA damage repair pathways were higher in lv-iPSCs and ESCs than in MEFs, and ionizing radiation did not substantially alter the expression of these genes (Fig. 3e). Thus the genomic instability of lv-iPSCs is unlikely to reflect changes in the expression level of genes involved in DNA damage repair.

### Weaker DNA damage repair response to ionizing radiation in lv-iPSCs

**Fig. 4:**
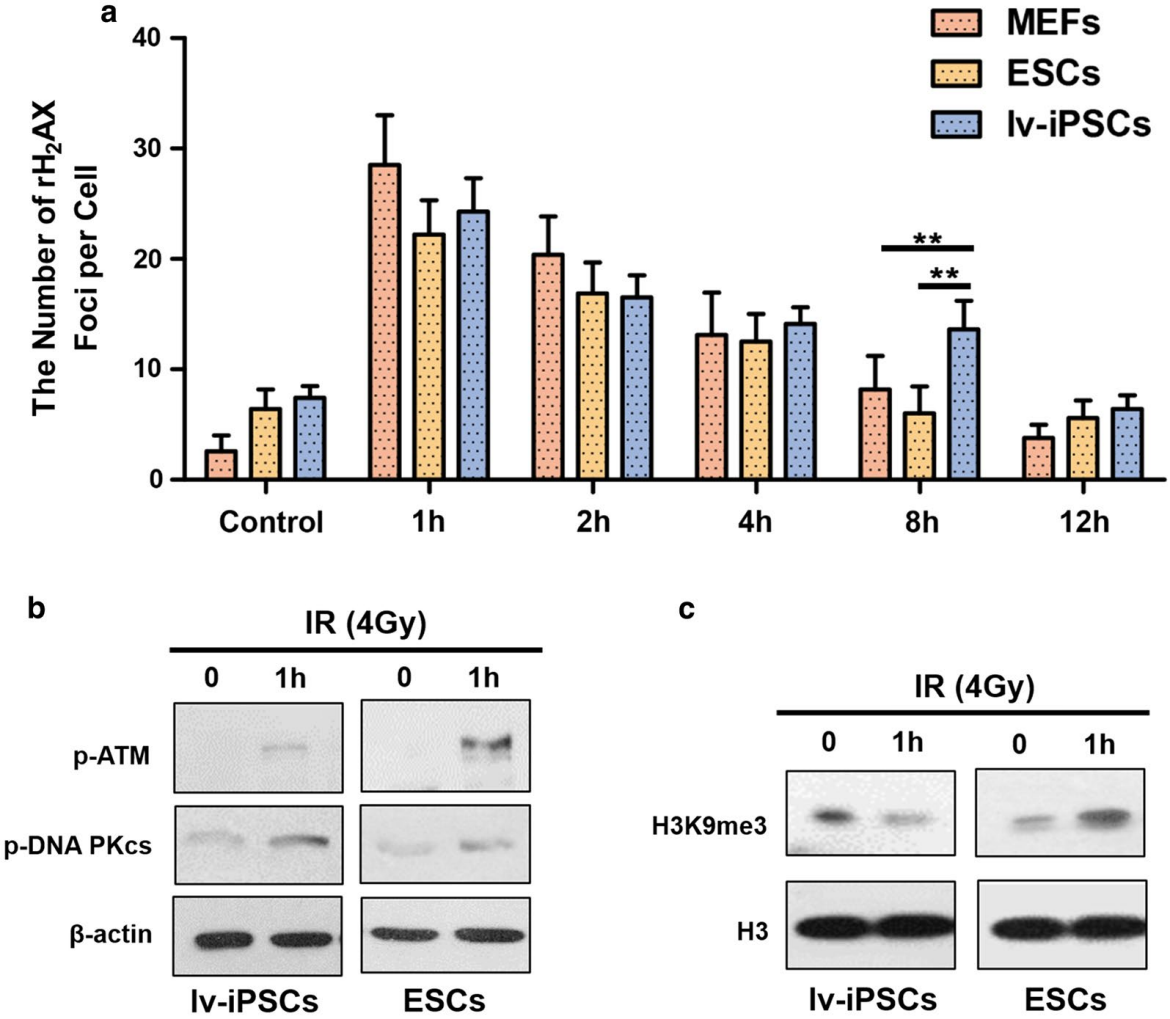
The phosphorylation level of DNA repair-associated proteins. **a** Quantification of the numbers of γ-H2AX foci in lv-iPSCs, ESCs and MEFs. Error bars represent the standard error of the mean (SEM) for the numbers of γ-H2AX foci per nucleus based on 4–5 fields, each containing approximately 20–30 cells. Significance of differences was assessed using Student’s *t* test. **P < 0.01 (three independent experiments). **b** Western blot analysis of phosphorylated ATM (p-ATM) and phosphorylated catalytic subunit of DNA protein kinase (p-DNA-PKcs) in lv-iPSCs and ESCs before and after ionizing irradiation. **c** Western blot analysis of the trimethylation level of H3K9 in lv-iPSCs and ESCs before and after ionizing irradiation

The phosphorylated histone variant H2AX (γ-H2AX) is a marker of double-strand breaks. Ionizing radiation significantly increased the number of γ-H2AX foci in lv-iPSCs, ESCs and MEFs, but the magnitude of decrease was much smaller in lv-iPSCs (Fig. 4a), suggesting lower capacity to repair DNA damage.

Next we tested whether the lower genomic stability of lv-iPSCs reflects deficiency in the error-free HR repair pathway. Indeed, we found ATM phosphorylation to be defective in lv-iPSCs (Fig. 4b) [30, 41]. We also found lower levels of H3K9me3, which recruits repair proteins to double-strand breaks, in irradiated lv-iPSCs than in irradiated ESCs or MEFs (Fig. 4c). All together, these findings may help explain the higher mutation rate of lv-iPSCs.

### Lower genomic stability in lv-iPSCs than ci-iPSCs

**Fig. 5:**
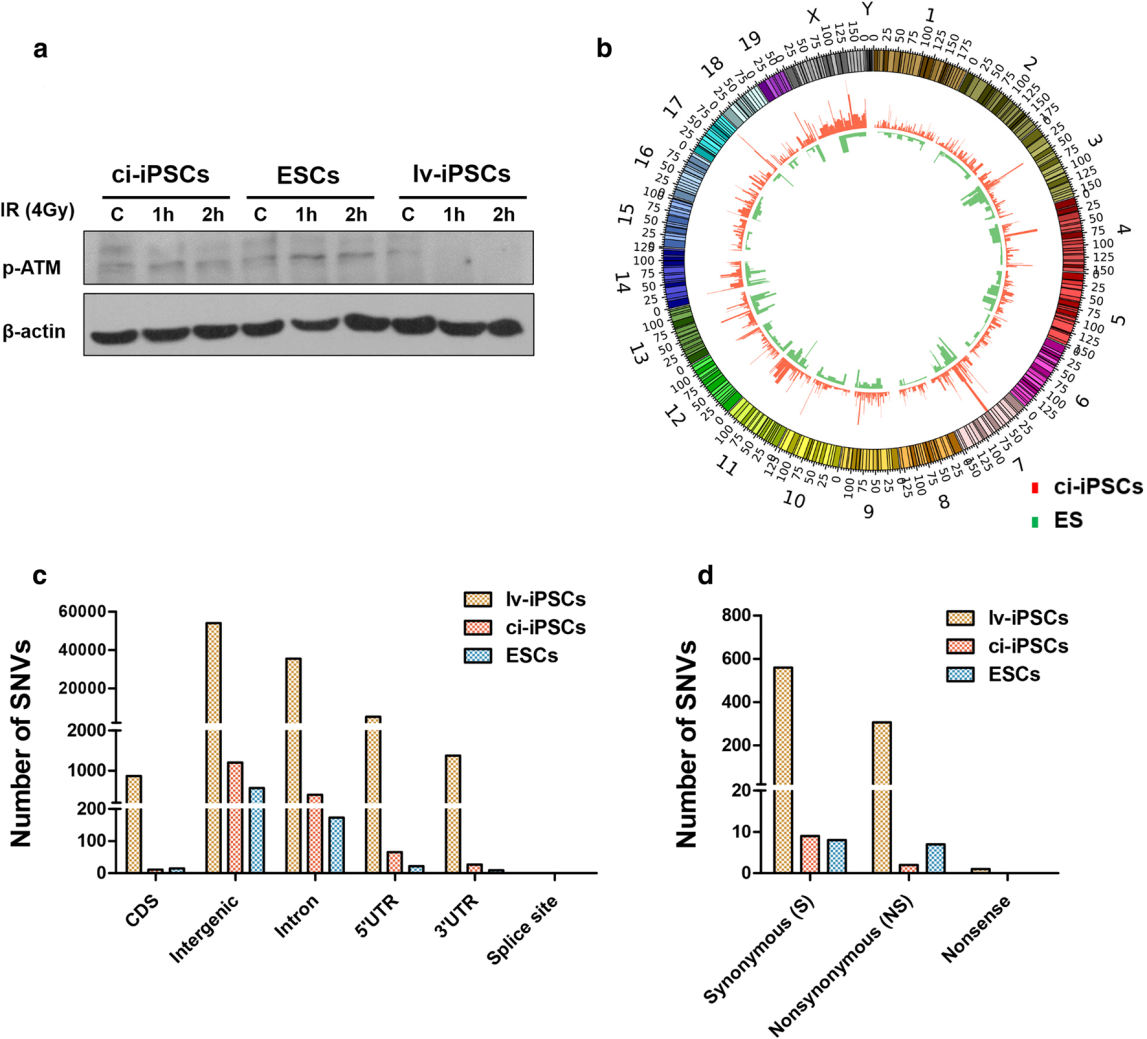
High genome stability of ci-iPSCs. **a** Western blot analysis of phosphorylated ATM (p-ATM) in ci-iPS, lv-iPS and ESCs before and after ionizing irradiation. **b** Circos plot showing genetic alterations in irradiated ci-iPSCs and ESCs, based on the corresponding untreated cells as a reference. Chromosome numbers are indicated as the outermost labels. **c** Histograms showing the number of SNVs in each genomic region of irradiated lv-iPSCs, ci-iPSCs and MEFs. *CDS* coding sequence, *SNV* single-nucleotide variants, *UTR* untranslated region. **d** Histograms showing the numbers of SNVs in the coding regions of irradiated lv-iPSCs, ci-iPSCs and MEFs

Treatment with ionizing radiation led to higher levels of phosphorylated ATM in ci-iPSCs than in lv-iPSCs (Fig. 5a). This may help explain the higher genomic stability of ci-iPSCs [41]. Whole-genome re-sequencing at 4 h after irradiation revealed 1709 SNVs in the ci-iPSCs; this was slightly more than in treated ESCs but far less than in lv-iPSCs (Fig. 5b). Similarly, the proportion of SNVs in coding sequences, introns, 5′ or 3′ UTRs and intergenic regions was slightly higher in ci-iPSCs than in ESCs, but much higher in lv-iPSCs (Fig. 5c, d). These results indicate greater genomic stability in ci-iPSCs than in lv-iPSCs, which is due at least in part to greater activity of the HR pathway of DNA damage repair.

### lv-iPSCs can tolerate more genomic DNA variation

**Fig. 6:**
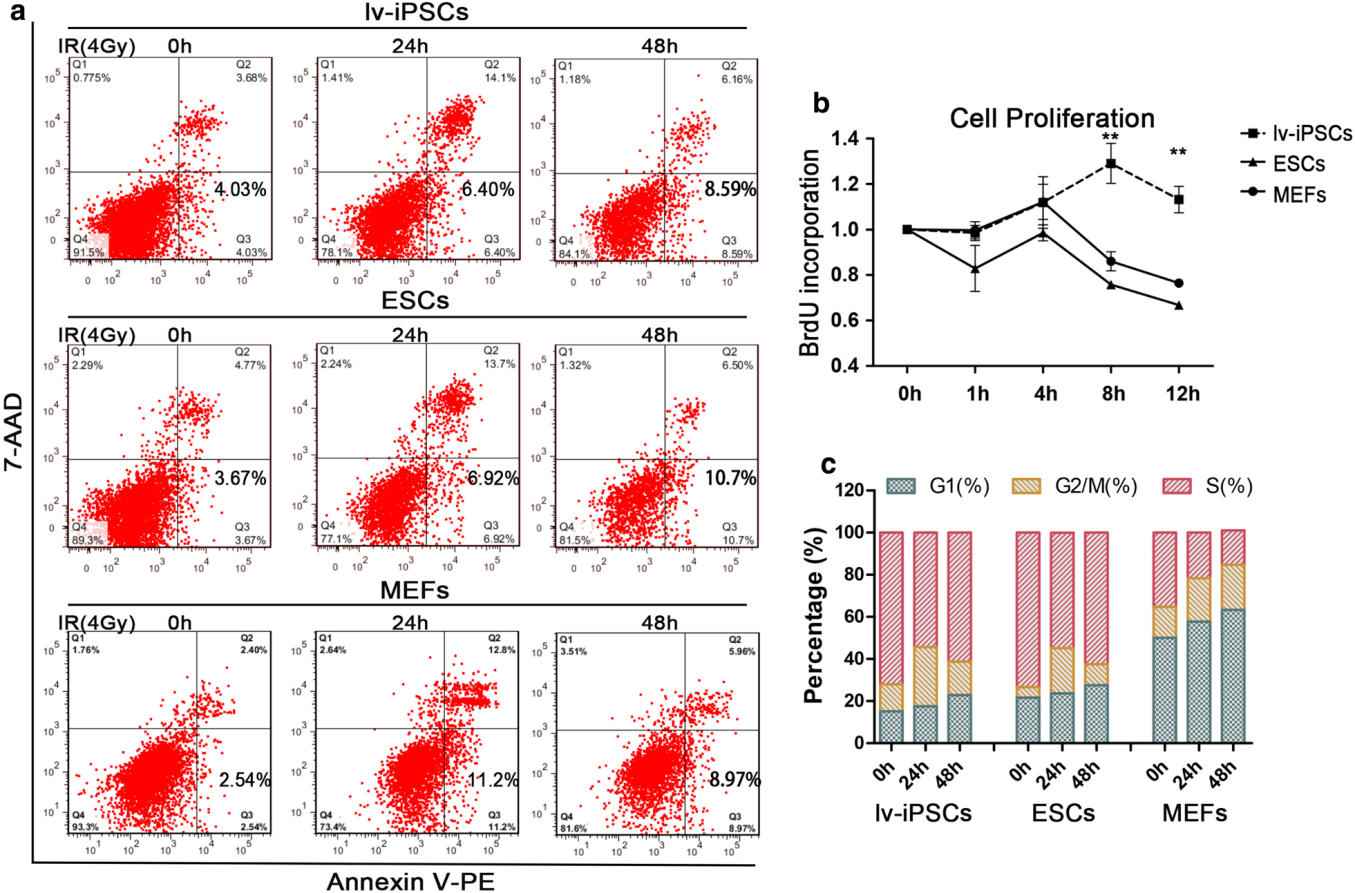
Greater tolerance of genomic DNA variation in lv-iPSCs. **a** Flow cytometric analysis of apoptosis rate in lv-iPSCs, ESCs and MEFs following ionizing irradiation (IR) for the indicated periods. *7-AAD* 7-amino-actinomycin. **b** Cell proliferation rate (based on BrdU incorporation) in lv-iPSCs, ESCs and MEFs exposed to ionizing irradiation for the indicated periods. Each point represents a mean of three replicates **P < 0.01. **c** Analysis of cell cycle distribution in lv-iPS, ESCs and MEFs exposed to ionizing radiation for the indicated periods

The abovementioned results led us to hypothesize that lv-iPSCs can survive with greater genomic variation than the other cell types. Consistent with this hypothesis, we found that lv-iPSCs indeed had more DNA variation than the other cell types, yet the percentage of apoptotic lv-iPSCs did not increase between 24 and 48 h after irradiation (Fig. 6a) and the rate of lv-iPSC proliferation was greater than that of ESCs or MEFs (Fig. 6b). When we analyzed whether irradiation arrested lv-iPSCs in the G2/M phase, we observed a high proportion of arrested cells at 24 h after irradiation, but a lower proportion at 48 h (Fig. 6c). We observed similar results with ESCs, showing an increased proportion of ESCs in G2/M phase at 24 h after irradiation and a lower radiation arrest at 48 h. These results suggest that lv-iPSCs tolerate greater genomic DNA variation than the other cell types.

### lv-iPSCs are more susceptible to DNA damage

**Fig. 7:**
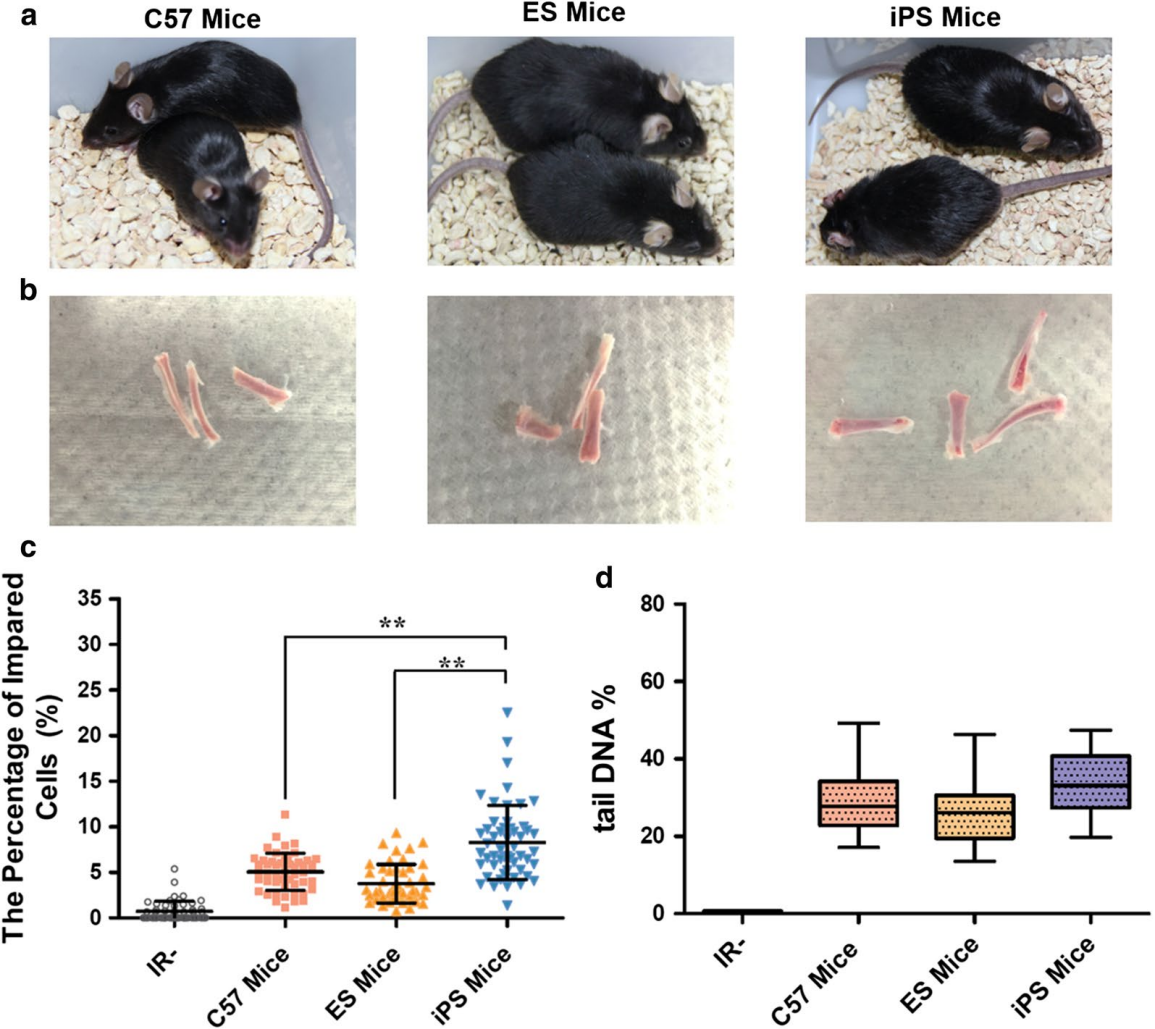
Genome stability of mice derived from lv-iPSCs or ESCs following exposure to ionizing radiation (IR). Controls were C57 mice. **a** Mice were generated from lv-iPSCs or ESCs through tetraploid embryo complementation. Representative results from three independent experiments are shown. **b** Examples of bones from the three types of mice, from which marrow cells were extracted. **c** Box plots showing the percentage of impaired bone marrow cells in each mouse strain. DNA damage was evaluated using single-cell gel electrophoresis **P < 0.01. **d** Box plots showing the percentage of Tail DNA in impaired cells as a measure of DNA damage. Tail DNA% = Tail DNA intensity/Cell DNA Intensity × 100%. CASP software was used to calculate tail moment based on 50–100 randomly selected cells per sample

Next we compared genomic stability in mice derived from lv-iPSCs versus ESCs. C57 mice were included as additional control. Irradiation of the mice led to a higher percentage of impaired bone marrow cells (Fig. 7a–c) and of tail DNA in bone marrow cells (Fig. 7d) in iPSC-derived mice than in ESC-derived mice and C57 mice. These results suggest that mice derived from lv-iPSCs have lower DNA damage repair capability than ESC-derived or C57 mice and are therefore more susceptible to DNA damage.

**Fig. 8:**
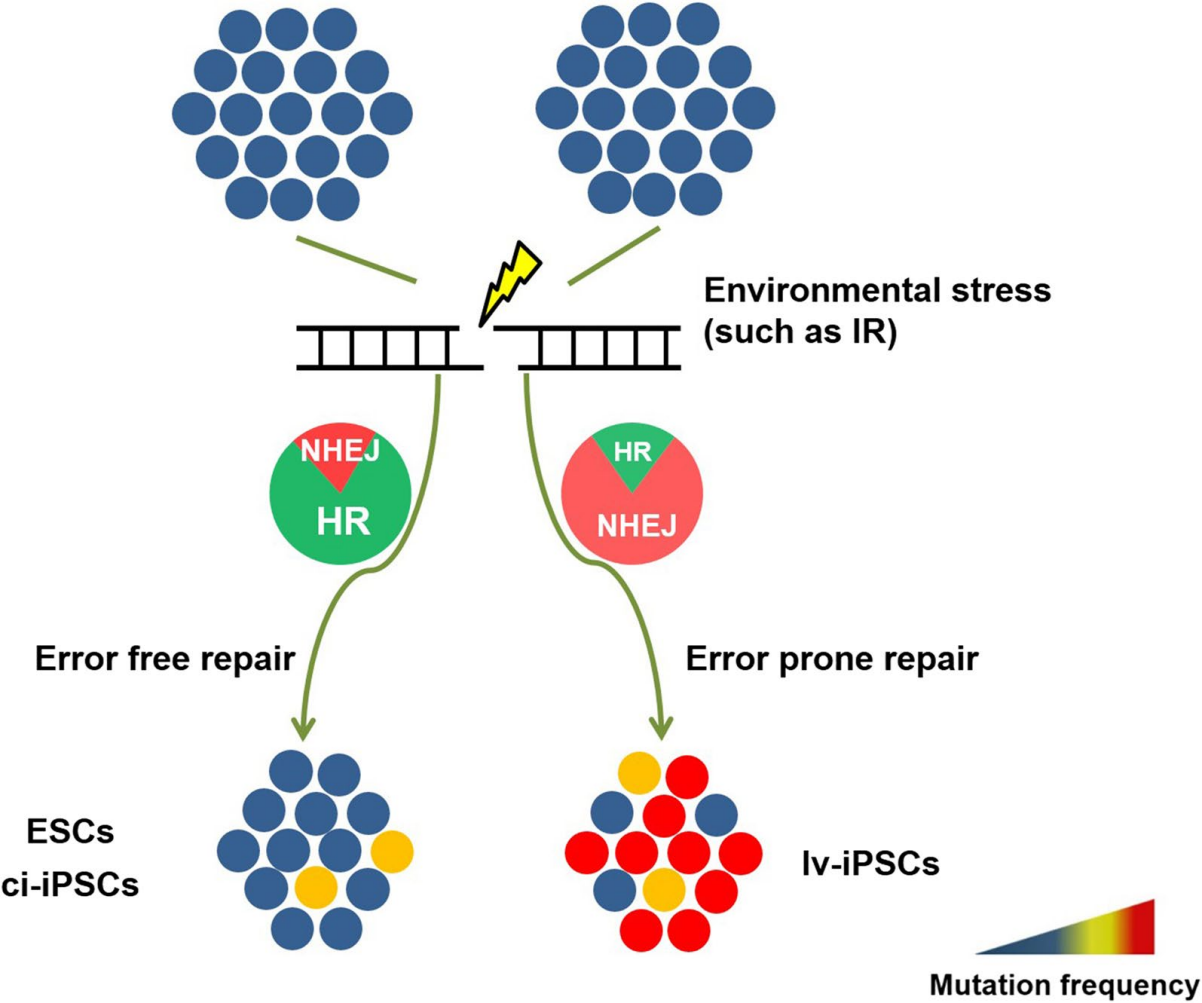
Diagram illustrating factors influencing the genome stability of iPSCs. Environmental factors contribute to genomic variations in lv-iPSCs. In response to double-strand DNA breaks, lv-iPSCs always adopt the error-prone NHEJ repair pathway. The resulting low fidelity of DNA repair makes the lv-iPSC genome unstable and the cells more vulnerable to environmental stress. Genomic stability of iPSCs appears to depend on the method used to generate them: ci-iPSCs show greater stability than lv-iPSCs. *HR* homologous recombination repair pathway, *IR* ionizing radiation, *NHEJ* non-homologous end joining

Taken together, our in vitro and in vivo experiments suggest that lv-iPSCs are more sensitive to environmental stress than ci-iPSCs, ESCs or MEFs. Ionizing radiation induces higher genomic mutation rates in lv-iPSCs, which nevertheless better tolerate the resulting genomic alterations. Genomic mutations that accumulate in lv-iPSCs are passed onto the next generation, resulting in genomic instability (Fig. 8).

## Discussion

Reprogramming to generate iPSCs more efficiently [29, 42–51] has been linked to the accumulation of genomic abnormalities [52–59]. This poses a problem for the use of iPSCs, since mice derived from such cells can tolerate the accumulation of somatic mutations for up to six generations [60]. In the present study, we used whole-genome sequencing to compare the genomic stability of iPSCs prepared using lentivirus or chemically, and to benchmark that stability against ESCs and MEFs. We found that ionizing irradiation led to the highest rate of somatic mutations and short indels in lv-iPSCs, and this correlated with low levels of ATM phosphorylation, indicating low fidelity of DNA damage repair [41]. Experiments in vitro and in mice derived from lv-iPSCs showed that this type of pluripotent cell tolerates genomic mutations better than the other cell types evaluated.

Although iPSCs resemble ESCs in morphology, gene expression profile and in vitro differentiation capacity, they differ substantially in genomic stability. The low fidelity of DNA repair observed in our study suggests that irradiation of lv-iPSCs induces a high rate of genomic abnormalities, which is less likely to trigger apoptosis in these cells and is therefore more likely to be tolerated, thus leading to a high rate of tumorigenesis in vivo. Compromised error-free HR pathway of DNA damage repair in lv-iPSCs may help explain the relatively high genomic instability in these cells. Indeed, inhibiting the HR pathway in iPSCs has been shown to destabilize the genome [61].

Our results suggest that the epigenetic status of iPSCs may contribute to, or modulate, their genomic instability. Variation in levels of H3K9me3 and phosphorylated ATM among iPSCs may mean that cells vary in their reliance on DNA damage repair pathways, which vary in their fidelity. Future studies should further examine the potential involvement of epigenetics and other factors in iPSC genomic instability.

Future work is also needed to clarify to what extent factors that are intrinsic or extrinsic to stem cells determine the risk of malignant transformation. Tomasetti et al. found that cancer risk in certain tissues correlated strongly with the number of divisions that the stem cells had undergone, suggesting that the accumulation of genomic mutations is primarily responsible for high risk of tumorigenesis [62]. Another study, in contrast, suggested that intrinsic factors account for only 10%–30% of cancer risk, with the majority of the risk due to extrinsic factors [63]. The results from the present study suggest that extrinsic factors induce more genomic mutations than intrinsic factors in lv-iPSCs. The high rate of tumorigenesis of iPSCs in vivo suggests that extrinsic factors strongly contribute to cancer risk and carcinogenesis.

## Conclusions

The present study demonstrated a low level of DNA damage repair in iPSCs. Ionizing radiation induced more somatic mutations and short indels in iPSCs than in ESCs or MEFs. Genome stability was higher in iPSCs induced chemically than in iPSCs induced with lentivirus. The high genome instability of lv-iPSCs appears to reflect increased NHEJ and decreased HR pathways of DNA damage repair, and could contribute to the high rate of tumorigenesis in vivo.
